# Glyoxalase 1−419C>A Variant Is Associated with Oxidative Stress: Implications in Prostate Cancer Progression

**DOI:** 10.1371/journal.pone.0074014

**Published:** 2013-09-10

**Authors:** Cinzia Antognelli, Letizia Mezzasoma, Ettore Mearini, Vincenzo Nicola Talesa

**Affiliations:** 1 Department of Experimental Medicine and Biochemical Sciences, University of Perugia, Perugia, Italy; 2 Department of Medical-Surgical Specialties and Public Health, University of Perugia, Perugia, Italy; IPO, Inst Port Oncology, Portugal

## Abstract

Glyoxalase 1 is a scavenging enzyme of potent precursors in reactive oxygen species formation and is involved in the occurrence and progression of human malignancies. Glyoxalase I A111E polymorphism has been suggested to influence its enzymatic activity. The present study was aimed at investigating the association of this polymorphism with oxidative stress and its implications in prostate cancer progression or survival. The polymorphism was genotyped in human differently aggressive and invasive prostate cancer cell lines, in 571 prostate cancer or 588 benign prostatic hyperplasia patients, and 580 healthy subjects by Polymerase Chain Reaction/Restriction Fragment Length Polymorphism. Glyoxalase 1 activity, the pro-oxidant Glyoxalase 1-related Argpyrimidine and oxidative stress biomarkers were evaluated by biochemical analyses. Glyoxalase 1 polymorphism was associated with an increase in Glyoxalase 1-related pro-oxidant Argpyrimidine and oxidative stress levels and cancer progression. The mutant A allele conferred a modest risk of prostate cancer, a marked risk of prostate cancer progression and a lower survival time, compared to the wild C allele. The results of our exploratory study point out a significant role for Glyoxalase 1 in prostate cancer progression, providing an additional candidate for risk assessment in prostate cancer patients and an independent prognostic factor for survival. Finally, we provided evidence of the biological plausibility of Glyoxalase 1 polymorphism, either alone or in combination with other ones, all related to oxidative stress control that represents a key event in PCa development and progression.

## Introduction

Glyoxalase 1 (GLO1) is a glutathione-dependent enzyme involved in the scavenging of Methylglyoxal (MG), a potent cytotoxic α-oxoaldehyde, with a strong ability to cross-link with protein amino groups, forming stable products called advanced glycation end products (AGEs) [1], [2]. Among them, argpyrimidine (AP) represents one of the major products deriving from MG modifications of proteins arginine residues [3], [4]. Both MG and AGEs are effective precursors of Reactive Oxygen Species (ROS) and free radical [5], [6], strongly implicated in cancer progression, including prostate cancer (PCa) [7]–[10]. A single nucleotide polymorphism (SNP), −419C>A (rs2736654), causing an Ala111Glu (A111E) substitution, has been identified in exon 4 of GLO1 gene [11] and associated with a decrease of GLO1 enzymatic activity [11]–[13]. GLO1 A111E polymorphism has been studied in some human disorders [11], [14]–[17], including cancer [18], [19], however, to the best of our knowledge, its role in PCa has never been investigated before. In the present study, we hypothesized that GLO1 polymorphism, linked to a decrease of GLO1 enzymatic activity, and, consequently, to an accumulation of GLO1-related pro-oxidative AP, could be associated with oxidative stress induction, thus counting as a novel mechanism in PCa progression. To this aim GLO1 polymorphism, AP and oxidative stress biomarkers (ROS, reduced glutathione, GSH, malonyldialdheyde, MDA) [20] levels were, firstly, studied in human differently aggressive and invasive LNCaP and PC3 prostate cancer cell lines, secondly, in urine sediments [21], [22] and blood of PCa and Benign Prostatic Hyperplasia (BPH) men, or healthy male age-matched subjects. Since our initial results pointed out a role for GLO1 in PCa progression, providing a biological background to the possible association of GLO1 A111E polymorphism with PCa, we, additionally, examined its association with progression, evaluated by stage and grade. A study on the association of GLO1 polymorphism and PCa risk and survival in selected subgroups was also performed. Besides, the evidence that PCa has an important genetic component is compelling from epidemiological, genetic and genome-wide association studies [23]–[26]. Some risk gene variants have been identified, which may predispose carriers to development and progression of such disease [24]; nevertheless, conflicting results have been obtained. Therefore, the study of other polymorphic genes is urgently needed. Enhancing the knowledge of how genetic factors influence PCa progression may improve the clinical management of such worldwide neoplasia. Finally, since we believe that single allelic variants themselves are not sufficient to predict, in many cases, the predisposition to the risk of PCa, we evaluated GLO1 A111E polymorphism in combination with other oxidative stress control-related polymorphic genes, that we previously found to be associated with the risk of PCa in a case/control study [27], whose BPH and PCa cohorts has been here included in the new enlarged populations. Additionally, the analysis was carried out in healthy male age-matched subjects.

The results of our exploratory study point out a significant role for GLO1 in PCa progression, providing an additional candidate for risk assessment in PCa patients and an independent prognostic factor for survival.

## Materials and Methods

### Cell Lines

Human prostate cancer LNCaP and PC3 cell lines were obtained from American Type Culture Collection (ATCC) and routinely maintained at 37°C in 5% CO_2_ in RPMI 1640 supplemented with 10% heat inactivated FBS, 1X L-glutamine, 1 mM sodium pyruvate, 1X non-essential amino acids, 100 units/ml of penicillin and 0.1 mg/ml of streptomycin (Invitrogen).

### Patients

A total of 1739 Caucasian Italian men were enrolled in this study. Of them, 1423 (PCa, BPH, healthy men) were enrolled between April 1999 and June 2005 from the Department of Urology (University of Perugia, Perugia, Italy), 316 (healthy men) were recruited between July 2005 and June 2013 from the same Department. The case group consisted of 571 patients with previously untreated, histologically verified PCa, diagnosed by transrectal ultrasound-guided biopsies. The indication for prostate biopsy was either a suspicious finding on digital rectal examination or elevated serum levels of Prostate Specific Antigen (PSA), or both. Disease was classified according to the World Health Organization criteria and staged according to the Tumour Node Metastasis (TNM) classification and the Gleason grading system (Gleason score). In particular, we stratified the analysis according to tumour stage (localized: T1/T2; locally advanced: T3/T4) and histological grade (low grade: Gleason 2–6 or well differentiated; moderate grade: Gleason 7 or moderately differentiated; high grade: Gleason 8–10 or poorly differentiated), according to the pathologic and/or radiologic reports. Evidence suggests that prostate tumours designated as Gleason score of 7 differ quite distinctly in regard to prognosis with those tumours with a Gleason score of 3+4 = 7 considered less aggressive than tumours with a Gleason score of 4+3 = 7 [28], [29]. Since, in preliminary analyses, we did not find any significant difference in GLO1 SNP association between the 3+4 or 4+3 tumour grades, we decided to gather them in a single group (Gleason score 7). The control group consisted of 588 age-, ethnicity- and geographically-matched individuals with BPH, whose diagnosis was based on lower-urinary tract symptoms, free uroflowmetry, evidence of increased prostate size (obtained by palpation or transrectal ultrasound) and in whom the presence of PCa was carefully excluded clinically and hystologically (negative biopsies for PSA >4 ng/ml, no T1a-T1b disease). For comparison, an healthy men group, including age-, ethnicity- and geographically-matched individuals, not suffering from any prostatic disease, was included in the study. The participation rates were 98%, 95% and 96% for the PCa, BPH and healthy men, respectively. All individuals who agreed to participate in the study were evaluated with a detailed questionnaire, which proved information about life-style and family history of cancer.

### Ethics Statement

All subjects gave written informed consent to the study, which was approved by the Committee on Bioethics of the University of Perugia in accordance with the principles established in the Helsinki declaration.

### GLO1−419C>A Polymorphism Genotyping

DNA from peripheral blood leukocytes was extracted with the NucleoSpin Blood Quick Pure Kit (Macherey-Nagel). GLO1-419C>A SNP detection was based upon Polymerase Chain Reaction-Fragment Length Polymorphism (PCR-RFLP) analysis as previously described [18], after PCR amplification. All genotyping was carried out by laboratory personnel blinded to case-control status of the samples, which included quality control samples for validation. Concordance for quality control samples was 100%.

### Oxidative Stress Control-related Gene Polymorphisms Genotyping

Single nucleotide polymorphisms (SNPs) for the oxidative stress control-related genes Glutathione S-transferase 1 (GSTP1 Ile>Val), Paraoxonase 1 (PON1-192Q>R, PON1-55 L>M) and cytochrome P450 17 (CYP17A1> A2) were detected as described by Antognelli et al. [27].

### Collection of Urine Samples After Prostate Massage

Collection of urine samples was performed as previously described [21], [22].

### Collection of Serum, Plasma and Lymphocytes from Blood Samples

Serum, plasma and lymphocytes were obtained from blood samples collected prior to rectal palpation, to exclude the possibility of influencing the results due to prostate manipulation. Serum and plasma were obtained according to standard protocols and used for GLO1 or MDA and GSH measurements, respectively. Lymphocytes were isolated in Lymphoprep separation medium (Axis-Shield, Oslo, Norway) [30] and used for ROS detection.

### GLO1 Enzymatic Specific Activity

Preparation of extracts from cell lines and GLO1 enzymatic specific activity were according to Antognelli et al. [31] and Mannervik et al. [32], respectively. Serum GLO1 estimation was performed as described by Chavan et al. [33].

### Western Blot

Whole-cell protein extraction and western blot were performed as previously described [31], [34]. An appropriate dilution of mouse anti-MG-AGE (Arg-Pyrimidine, AP) mAb (Antibodies-online, GmbH) and anti-β-actin mAb (Santa Cruz Biotechnology) were used. AP measurement in plasma was performed according to Raj et al. [35].

### Measurement of Oxidative Stress Biomarkers (ROS, GSH and MDA)

ROS and GSH were detected according to Szabados et al. [36] or Guzel et al [37], respectively. MDA was measured using OxiSelect™ TBARS Assay Kit (MDA Quantitation) (Cell Biolabs).

### Statistical Analysis

Analysis of data was performed using SPSS 11.0 for Windows. Descriptive analysis included determination of standard deviation (SD) as well as Student’s t-test. GLO1 serum levels were log-transformed as the subgroups (localized, locally advanced or low, intermediate and high grade) were not showing normal distribution of the data as per 1 sample-KS test. Distribution of metabolites was normal in each of the above mentioned subgroups. The χ^2^ test was used to compare genotype/allele frequencies between PCa cases and BPH or healthy subjects controls. Odds ratio (ORs) was calculated to estimate the Relative Risk for PCa by unconditional logistic regression analysis and adjusted for potential modifying factors, including age (continuous) and Prostate-specific antigen (PSA, continuous). Allele frequencies were calculated by direct gene counting. χ^2^ test was used for testing of Hardy-Weinberg equilibrium (HWE). GLO1 SNP was tested for possible effects on oxidative stress control-related traits by linear regression analysis under an additive model, incorporating covariates of age and PSA. Survival analysis of selected PCa cases was used to test the possible effect of genotypes on the risk of dying from PCa. For this purpose survival curves were constructed using the Kaplan-Meier method, and the differences between the groups were tested by the log-rank method. The multivariate analysis of the probable prognostic factors for survival was performed using Cox’s proportional hazard regression analysis. The relative hazard ratios (HR) with 95% confidence intervals were assessed adjusting for potential confounding variables, so considered if they changed the HRs of any of the genetic variants by at least 5%. Survival time was calculated from the dates of PCa diagnosis to the date of PCa-specific death, collected from the databases of the Regional Cancer Registry in 2010. Adjustment for multiple testing was performed using permutation-based methods [38], [39]. For all results, a p-value of less than 0.05 was regarded as statistically significant.

## Results

### Characteristics of PCa Cases and BPH or Healthy Controls

The main characteristics of PCa cases and BPH or healthy controls are presented in Table 1. The cases and controls appeared well matched on age, Body Mass Index (BMI), drinking or smoking status and physical activity (p>0.05). Serum PSA levels were significantly higher in PCa compared to BPH or healthy men (p<0.0001). When stratified according to the clinical stage, 52.0% and 48.0% of the patients had localized (T1/T2) and locally advanced (T3/T4) disease, respectively. When stratified according to tumor grade (Gleason score), 44.7%, 28.2% and 27.1% of the patients had low (2–6), moderate (7) and high (8–10) grade disease, respectively.

**Table 1:** Demographic and clinical data.

Variables	PCa (n = 571)	BPH (n = 588)	Healthy men (n = 580)	p value
**Age at diagnosis, yr (mean ± SD)**	68 (±5)	69 (±6)	66 (±7)	>0.05*
**PSA, ng/mL (mean ± SD)**	44.8 (±11.4)	10.0 (±8.3)	8.0 (±6.6)	<0.0001*
**Tumor Stage, n (%)**				
Localized	297 (52.0)	N/A	N/A	N/A
Locally advanced	274 (48.0)	N/A	N/A	N/A
**Gleason score, n (%)**				
Low grade	255 (44.7)	N/A	N/A	N/A
Moderate grade	161 (28.2)	N/A	N/A	N/A
High grade	155 (27.1)	N/A	N/A	N/A
**BMI (kg/m^2^), n (%)**				
<25	340 (59.5)	367 (62.4)	354 (61.0)	NS
25–30	211 (37.0)	206 (35.0)	215 (37.1)	NS
>30	20 (3.5)	15 (2.6)	11 (1.9)	NS
**Physical activity**				
Moderate	512 (89.7)	516 (87.8)	512 (88.3)	NS
Exhaustive	59 (10.3)	72 (12.2)	68 (11.7)	NS
**Current alcohol intake**				
Never	88 (15.4)	110 (18.7)	121 (20.9)	NS
Once a month or less	160 (28.0)	177 (30.1)	167 (28.8)	NS
Daily-weekly	323 (56.6)	301 (51.2)	292 (50.3)	NS
**Smoking status**				
Non-smokers^a^	320 (56.0)	312 (53.1)	326 (56.2)	NS
Ex-smokers^b^	103 (18.0)	120 (20.4)	113 (19.5)	NS
Current smokers^c^	148 (26.0)	156 (26.5)	141 (24.3)	NS

PCa = prostate cancer; BPH = benign prostatic hyperplasia

* PCa versus BPH or healthy controls by Student’s t test; N/A = not applicable; PSA = prostate specific antigen, BMI = body mass index;

a never smokers;

b tobacco consumption stopped from 6.5±3.2 years with a previous average tobacco consumption estimated at 45 pack-years (range, 18–104);

c with an average tobacco consumption estimated at 39 pack-years (range, 16–196). NS = not significant by χ^2^ test for categorical variables.

### Association of GLO1 Polymorphism with Oxidative Stress and PCa Progression

**Figure 1:**
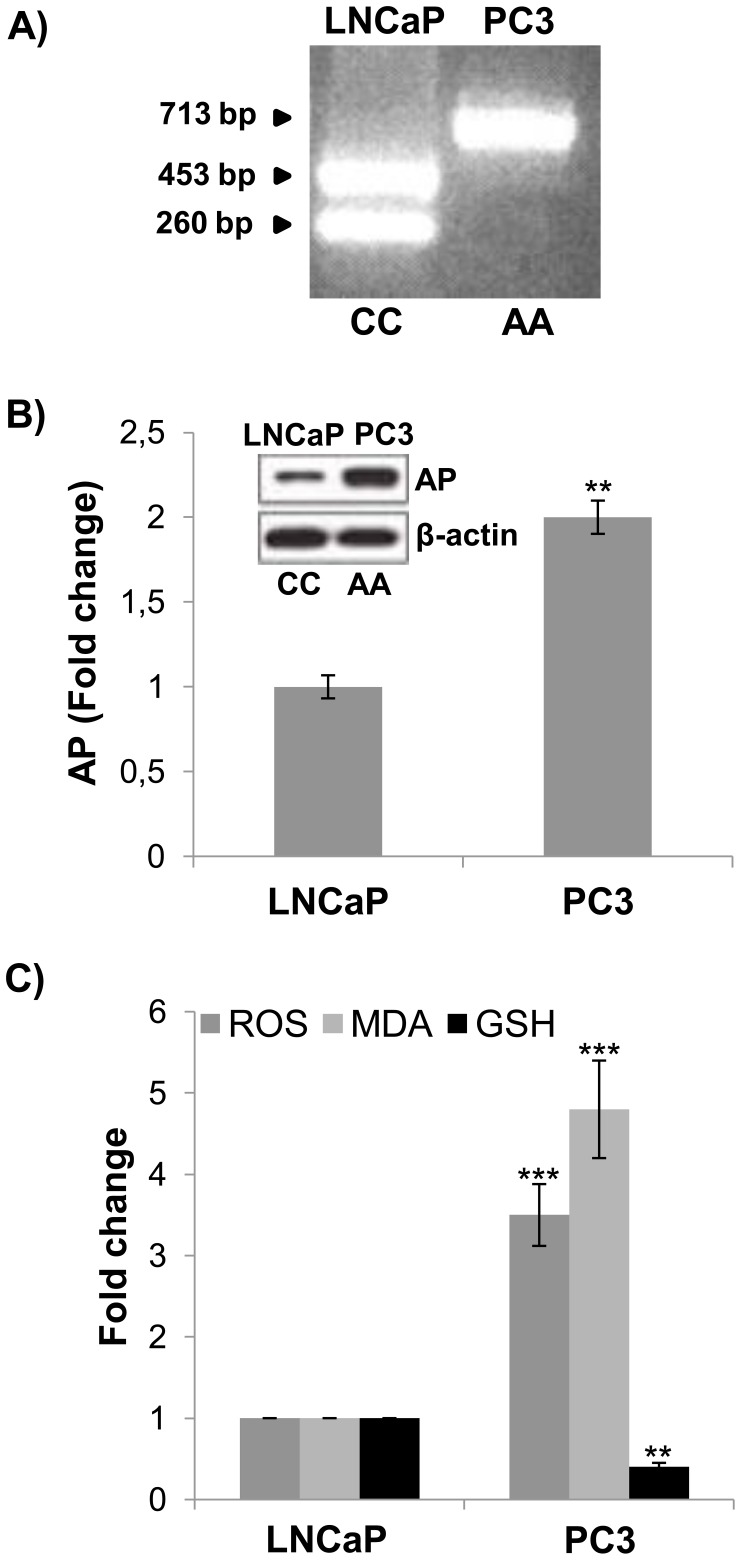
GLO1−419C>A polymorphism genotyping, argpyrimidine and oxidative stress indices in LNCaP and PC3 cells. (A) GLO1 −419C>A homozygous wild type (CC) LNCaP and homozygous mutant type (AA) PC3 cells; (B) Argpyrimidine (AP) intracellular levels and densitometric analysis from Western blot detection. Western blot was obtained by using a mAb mouse anti-AP. The blot was stripped of the bound Ab and re-probed with mouse anti-β-actin, to confirm equal loading. The Western blot shown is representative of three separate experiments. (C) Reactive oxygen species (ROS), malondialdehyde (MDA) and reduced glutathione (GSH) intracellular levels. Histograms indicate means ± SD of three different cultures each of one was tested in quadruplicate and expressed as fold change.**P<0.001, **P<0.001.

In order to study the association of GLO1 A111E polymorphism with oxidative stress, we, firstly, studied GLO1 genotypes and enzymatic specific activity in two differently aggressive and invasive human prostate cancer cell lines. We found that poorly aggressive, less invasive and androgen-dependent LNCaP cells were homozygous for C allele (Figure 1A) and had a GLO1 specific enzymatic activity significantly (p<0.03) higher (mean ± SD, 1.47±0.025 µmol/min/mg protein) than highly aggressive, more invasive and androgen-insensitive PC3 cells (mean ± SD, 0.84±0.01 µmol/min/mg protein), omozygous for A allele (Figure 1A). Since GLO1 is required for MG detoxification that is, intracellularly, rapidly converted into AGEs [1], we then analyzed intracellular levels of AP, the major AGE derived from spontaneous MG adduction of arginine residues [3], [4], [40], finding that in PC3 cells, harboring the Ala isoform of GLO1, they were significantly (p<0.001) doubled compared to LNCaP cells, expressing the Glu isoform (Figure 1B). Finally, since AP has been shown to be an effective precursor of ROS and free radical production [5], [6], [41], we analyzed the intracellular levels of oxidative stress biomarkers, finding a significant increase in MDA and ROS or decrease in GSH intracellular levels in PC3 cells compared to LNCaP (Figure 1C). Since these *in vitro* results appeared to support the potential association of GLO1 polymorphism with progression, we performed measurements of the same parameters in blood samples and cells from urine sediments of patients with localized and locally advanced or low, intermediate and high grade PCa. GLO1 activity, AP and oxidative stress indices, both in blood or cells from urine sediments, were significantly different among PCa patients stratified by stage and grade, carrying CC, CA and AA genotypes of GLO1 polymorphism (all p<0.05) (Table 2), reflecting the trend obtained in the studied differently aggressive cell lines.

**Table 2:** Association between GLO1 −419C>A polymorphism and metabolite levels in prostate cancer progression evaluated by stage and grade.

	Blood samples					Cells from urine sediments				
	Localized (n = 297)					Localized (n = 297)				
	GLO1	AP	MDA	GSH	ROS	GLO1	AP	MDA	GSH	ROS
**CC**	2.39±0.10	94.40±4.20	2.51±0.10	8.91±0.41	0.39±0.03	1.32±0.10	93.92±5.90	2.49±0.09	8.82±0.46	0.37±0.05
**CA**	2.15±0.08	107.01±2.7	2.83±0.04	7.92±0.15	0.59±0.02	1.18±0.02	106.75±1.2	2.82±0.02	7.97±0.15	0.56±0.02
**AA**	1.84±0.10	118.60±2.1	3.21±0.12	7.12±0.11	0.77±0.03	1.02±0.03	119.30±3.2	3.18±0.06	7.00±0.55	0.76±0.02
	**Locally advanced (n = 274)**					**Locally advanced (n = 274)**				
	**GLO1**	**AP**	**MDA**	**GSH**	**ROS**	**GLO1**	**AP**	**MDA**	**GSH**	**ROS**
**CC**	2.13±0.11	98.47±8.61	2.67±0.11	10.25±0.23	0.42±0.02	1.30±0.10	97.96±6.21	2.62±0.12	10.19±0.21	0.44±0.02
**CA**	1.85±0.07	120.54±3.10	3.14±0.01	8.22±0.10	0.67±0.01	1.03±0.04	122.55±3.4	3.11±0.08	7.7±0.01	0.66±0.02
**AA**	1.53±0.05	141.68±4.61	3.74±0.03	5.56±0.11	0.89±0.01	0.67±0.07	141.95±5.5	3.69±0.11	5.7±0.02	0.82±0.01
	**Low grade (n = 255)**					**Low grade (n = 255)**				
	**GLO1**	**AP**	**MDA**	**GSH**	**ROS**	**GLO1**	**AP**	**MDA**	**GSH**	**ROS**
**CC**	2.37±0.17	92.78±10.00	2.60±0.02	9.21±1.2	0.41±0.01	1.29±0.10	91.87±2.32	2.57±0.04	9.18±1.3	0.39±0.04
**CA**	2.00±0.10	117.12±1.2	2.79±0.01	7.86±0.05	0.60±0.02	1.14±0.04	113.88±2.3	2.79±0.06	7.40±0.09	0.60±0.01
**AA**	1.48±0.10	135.68±2.4	3.04±0.01	5.94±0.16	0.82±0.01	0.98±0.02	130.49±3.1	3.03±0.06	5.00±0.10	0.78±0.02
	**Moderate grade (n = 161)**					**Moderate grade (n = 161)**				
	**GLO1**	**AP**	**MDA**	**GSH**	**ROS**	**GLO1**	**AP**	**MDA**	**GSH**	**ROS**
**CC**	2.20±0.11	95.01±7.10	2.65±0.07	8.94±0.23	0.43±0.03	1.25±0.11	94.08±7.38	2.63±0.04	8.90±0.21	0.40±0.01
**CA**	1.65±0.05	124.36±4.0	3.05±0.03	6.57±0.01	0.70±0.01	1.02±0.03	123.66±6.1	3.01±0.01	6.54±0.02	0.68±0.01
**AA**	1.25±0.08	163.83±5.8	3.53±0.07	3.85±0.01	0.98±0.01	0.70±0.02	161.45±5.2	3.49±0.01	3.80±0.03	0.94±0.02
	**High grade (n = 155)**					**High grade (n = 155)**				
	**GLO1**	**AP**	**MDA**	**GSH**	**ROS**	**GLO1**	**AP**	**MDA**	**GSH**	**ROS**
**CC**	2.10±0.15	98.21±11.11	2.68±0.03	8.78±0.69	0.43±0.06	1.20±0.17	97.25±11.43	2.68±0.06	8.71±0.61	0.43±0.08
**CA**	1.47±0.08	144.30±6.3	3.33±0.10	6.12±0.09	0.77±0.01	0.89±0.01	143.28±5.10	3.29±0.09	6.20±0.12	0.74±0.01
**AA**	0.93±0.06	193.90±4.2	3.87±0.06	3.30±0.07	1.07±0.01	0.53±0.01	192.14±2.30	3.97±0.07	3.00±0.05	1.04±0.01

GLO1(glyoxalase 1): µmol/min (log-transformed); AP (argpyrimidine): pmol/10 µmol protein; MDA (malonyldialdheyde): nmol/mL; GSH (reduced glutathione): nmol/mg protein; ROS (reactive oxygen species): expressed as rhodamine 123 fluorescence level. p values are calculated using linear regression under an additive model, incorporating age and PSA as covariates; p<0.05.

### GLO1 Polymorphism and PCa Progression

Table 3 reports the effects of GLO1 polymorphism on PCa progression evaluated by clinical stage and histological grade. Individuals carrying at least one variant A allele (CA+AA) had a moderately higher risk of developing localized PCa (OR = 1.57, 95% CI = 1.17–2.11, p = 0.002) while a strong association was found with locally advanced PCa stage (OR = 8.97, 95% CI = 5.77–14.02, p<0.0001), compared with individuals with CC genotype. Similarly, individuals carrying at least one variant A allele (CA+AA) had a moderately higher risk of developing low grade PCa (OR = 1.46, 95% CI = 1.07–1.98, p = 0.013) while a strong association was found with moderate and high grade PCa (OR = 7.20, 95% CI = 4.28–12.20, p<0.0001 and OR = 10.28, 95% CI = 5.64–19.05, p<0.0001, respectively), compared with individuals with CC genotype.

**Table 3:** Association between GLO1 −419C>A polymorphism and the progression of prostate cancer (PCa) evaluated by stage and grade.

Categories			Crude			Adjusted*		
	CC	CA+AA	OR	95% CI	p value	OR	95% CI	p value
**BPH, n = 588 (%)**	297 (50.5)	291 (49.5)	1.00	(reference)		1.00	(reference)	
**Healthy men, n = 580 (%)**	297 (51.2)	283 (48.8)	1.00	(reference)		1.00	(reference)	
**PCa, n = 571**								
**Clinical stage**								
**Localized, n = 297 (%)**	117 (39.4)	180 (60.6)	1.57	1.17–2.11	= 0.002	1.58	1.18–2.13	= 0.002
			1.61^a^	1.20–2.17	= 0.001	1.60	1.19–2.15	= 0.001
**Locally advanced, n = 274 (%)**	28 (10.2)	246 (89.8)	8.97	5.77–14.02	<0.0001	8.96	5.75–13.99	<0.0001
			9.22^a^	5.93–14.42	<0.0001	9.23	5.96–14.45	<0.0001
**Grade**								
**Low grade, n = 255 (%)**	105 (41.2)	150 (58.8)	1.46	1.07–1.98	= 0.013	1.45	1.06–1.97	= 0.012
			1.50^a^	1.10–2.04	<0.01	1.51	1.12–2.05	<0.01
**Moderate grade, n = 161 (%)**	20 (12.4)	141 (87.6)	7.20	4.28–12.20	<0.0001	7.21	4.30–12.23	<0.0001
			7.40^a^	4.40–12.55	<0.0001	7.38	4.38–12.51	<0.0001
**High grade, n = 155 (%)**	14 (9.0)	141 (90.9)	10.28	5.64–19.05	<0.0001	10.30	5.65–19.07	<0.0001
			10.57	5.80–19.60	<0.0001	10.58	5.81–19.62	<0.0001

BPH = benign prostatic hyperplasia controls; CI = Confidence Interval; OR = Odds Ratio *for age and Prostate Specific Antigen (PSA) in logistic regression model.^ a^compared with the healthy men group.

### GLO1 Polymorphism and PCa Risk

A study on the association of GLO1 polymorphism and PCa risk was also performed.The genotype and allele frequencies of GLO1 −419C>A polymorphism in PCa cases and BPH or healthy controls are shown in Table 4. The genotype frequencies in BPH and PCa patients or healthy men all conformed to HWE (p>0.05). We found that the prevalence of the wild type CC genotype was lower in PCa (43.3%) than in BPH (50.5%) or healthy men (52.2%) population, and that the frequency of CA genotype was higher among the PCa (46.2%) than among BPH (41.7%) or healthy men (40.7%) group. The prevalence of the mutant AA genotype was higher among the PCa (10.5%) compared to BPH (7.8%) or healthy men (7.1%) group. The difference in the frequencies distribution of C and A allele among PCa and BPH patients or healthy men was also significant (p = 0.010). Several studies reported that the frequencies of the mutant A allele in different normal populations worldwide showed values between 27.2% and 51.7% [11], [17], [18], [42], [43]. Our results on A allele frequency in the BPH or healthy men groups were also in the reported range. Since no significant differences were observed between healthy and BPH men, BPH was chosen as the reference group. Individuals with CA and AA genotypes had a marginal but significant increased susceptibility to PCa occurrence, compared with individuals with the CC genotype (OR = 1.34, 95% CI = 1.06–1.70, p = 0.013; OR = 1.57, 95% CI = 1.01–2.44, p = 0.035, respectively). Furthermore, the variant A allele was also associated with a modest significant increased risk for PCa, compared with the C allele (OR = 1.26, 95% CI = 1.05–1.51, p = 0.010).

**Table 4:** Genotype and allele frequencies of GLO1 −419C>A polymorphism among prostate cancer (PCa) cases and benign prostatic hyperplasia (BPH) or healthy controls and the associations with the risk of Pca.

Genotype−419C>A	PCa (n = 571) (%)	BPH (n = 588) (%)	Healthy men (n = 580) (%)	Crude			Adjusted*		
				OR	95% CI	p value	OR	95% CI	p^a^ value
**CC**	247 (43.3)	297 (50.5)	303 (52.2)	1.00	(reference)		1.00	(reference)	
**CA**	264 (46.2)	245 (41.7)	236 (40.7)	1.30	1.01–1.66	0.036	1.29	1.00–1.65	0.035
**AA**	60 (10.5)	46 (7.8)	41 (7.1)	1.57	1.01–2.44	0.035	1.56	1.00–2.43	0.034
**CA+AA**	324 (56.7)	291 (49.5)	277 (47.7)	1.34	1.06–1.70	0.013	1.35	1.07–1.71	0.014
**C allele**	758 (66.4)	839 (71.3)	842 (71.5)	1.00	(reference)		1.00	(reference)	
**A allele**	384 (33.6)	337 (28.7)	318 (27.8)	1.26	1.05–1.51	0.010	1.25	1.06–1.52	0.011

* for age and PSA in logistic regression model. CI = Confidence Interval; OR = Odds Ratio.

a No significant differences were observed between healthy and BPH men, therefore BPH was chosen as the reference group.

### GLO1 Polymorphism and PCa Survival in Selected Subgroups by Tumor Stage and Grade

**Figure 2:**
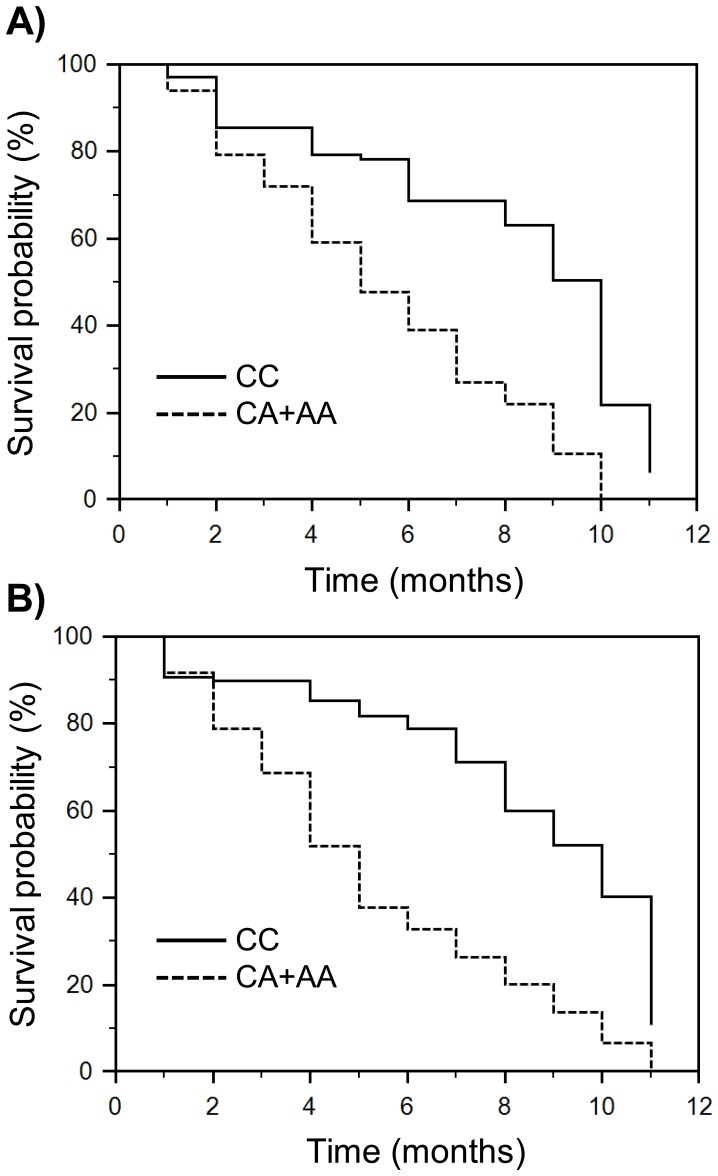
Kaplan-Meier survival curves for localized or low grade prostate cancer patients subgroups by GLO1–419C>A. The individuals with the variant CA/AA genotypes showed significantly lower survival rates than individuals with the CC genotype in both localized (A) or low grade (B) subgroups.

Since a significant percentage of patients with localized (60.6%, Table 3) or low grade (58.8%, Table 3) PCa contained the risk A allele, we hypothesized that in these groups, patients carrying the variant A allele might be at a higher risk of a later tumor progression, compared to patients carrying the CC. When the log-rank and the Cox’s regression analysis were used to assess the associations between GLO1 polymorphism and survival time, individuals with the CA/AA genotypes showed a lower survival time than the individuals with the CC genotype in both localized or low grade subgroups (Table 5, Figure 2).

**Table 5:** Association between GLO1 −419C>A genotypes and prostate cancer (PCa) survival in selected subgroups by tumor stage and grade.

Genotype	Patients (n)	Deaths (n)	MST (years)	Crude HR	95% CI	p value	HR^a^	95% CI	p value
**Localized (T1/T2)**									
(n = 297)									
**CC**	117	78	10.0	1.00			1.00		
**CA+AA**	180	176	5.0	2.53	1.98–3.24	<0.0001	2.48	(1.91–3.19)	<0.0001
**Low grade (GS 2–6)**									
(n = 255)									
**CC**	105	67	10.0	1.00			1.00		
**CA+AA**	150	147	5.0	2.38	1.82–3.11	<0.0001	2.35	(1.79–3.08)	<0.0001

CI = Confidence Interval; HR = hazard ratio; ^a^adjusted by age and prostate specific antigen (PSA). MST = median survival time.

In addition, the multivariate analysis used to delineate significant prognostic factors for survival, showed in both subgroups that the risk A allele was an independent prognostic factor for survival after adjustment for age and PSA (localized PCa: adjusted HR = 2.48, 95% CI: 1.91–3.19; low grade PCa: adjusted HR = 2.35, 95% CI: 1.79–3.08) (Table 5).

### Combination of GLO1, GSTP1, PON1-192, PON-1 5, CYP17 Polymorphisms as Predictive Factor for PCa and Association with Oxidative Stress

In a case/control study whose cohorts has now been included in the present enlarged populations, we previously found that polymorphisms in oxidative-stress control-related GSTP1, PON1-192, PON-1 55 and CYP17 genes were associated with the risk of PCa. In that work, no association of the same gene polymorphisms was found with tumor progression or survival [27]. Here, we firstly confirmed the previously observed results integrating them with a comparison with an healthy men group (Table 6). Secondly, since we believe that the combination of more polymorphisms in each single case, with respect to a single one, may be a more predictive factor for the association to the risk of PCa, we evaluated such a combination, also including GLO1 polymorphism. Table 7 shows that the detection of the GSTP1Ile/Ile-PON1/192RR-PON1/55LL-CYP17A2A2-GLO1CC or GSTP1Ile/Ile-PON1/192QQ-PON1/55LM-CYP17A1A2-GLO1CA or GSTP1Ile/Val-PON1/192QR-PON1/55MM-CYP17A1A1-GLO1AA genotype combination at individual level, might lead to the identification of patients with low, intermediate and high risk for PCa, respectively. A significant (p<0.05) association of the Low, Intermediate and High risk genotypes with the levels of ROS, GSH and MDA oxidative stress biomarkers in blood and cells from urine sediments was observed (Table 7).

**Table 6:** Prevalence of GSTP1, PON1/192, PON1/55 and CYP17 polymorphisms and risk of prostate cancer (Pca).

Genotype	PCa (n = 571) (%)	BPH (n = 588) (%)	Healthy men (n = 580) (%)	Crude			Adjusted*		
				OR	95% CI	p value	OR	95% CI	p value
**GSTP1**									
w/w	255 (44.6)	357 (60.7)	375 (64.7)	1.00	(reference)^a^		1.00	(reference)^a^	
w/m	285 (50.0)	194 (33.0)	176 (30.3)	2.06	1.60–2.64	<0.0001	2.03	1.62–2.65	<0.0001
m/m	31 (5.4)	37 (6.3)	29 (5.0)	1.17	0.69–2.00	0.534	1.15	0.65–2.06	0.538
**PON1/192**									
w/w	291 (51.0)	347 (59.0)	360 (62.1)	1.00	(reference)^a^		1.00	(reference)^a^	
w/m	250 (43.8)	136 (23.1)	122 (21.0)	2.19	1.68–2.87	<0.0001	2.16	1.66–2.84	<0.0001
m/m	30 (5.2)	105 (17.9)	98 (16.9)	0.34	0.22–0.54	<0.0001	0.36	0.25–0.55	<0.0001
**PON1/55**									
w/w	180 (31.5)	241 (41.0)	256 (44.1)	1.00	(reference)^a^		1.00	(reference)^a^	
w/m	291 (51.0)	273 (46.4)	267 (46.0)	1.43	1.10–1.86	0.006	1.45	1.11–1.89	0.005
m/m	100 (17.5)	74 (12.6)	57 (9.8)	1.81	1.25–2.63	0.001	1.78	1.29–2.66	0.001
**CYP17**									
w/w	251 (44.0)	195 (33.2)	202 (34.8)	3.18	2.16–4.69	<0.0001	3.21	2.19–4.71	<0.0001
w/m	267 (46.7)	262 (44.5)	246 (42.4)	2.52	1.73–3.68	<0.0001	2.54	1.74–3.69	<0.0001
m/m	53 (9.3)	131 (22.3)	132 (22.8)	1.00	(reference)^a^		1.00	(reference)^a^	

w: wild allele, m: mutant allele; *for age and PSA in logistic regression model. CI = confidence interval; OR = odds ratio.

a Comparable results were observed between healthy and BPH men, therefore BPH was chosen as the reference group.

**Table 7:** Risk level according to genotype combination and concentration of oxidative stress biomarkers in blood and cells from urine sediments according to patients’ class of risk.

Genotype	PCa (n)	BPH (n)	Crude					Adjusted*				
			OR		95% CI		p value	OR		95% CI		p value
**Low risk**	765	1131	1.00		(reference)			1.00		(reference)		
**Intermediate risk**	1368	1484	1.36		1.21–1.54		<0.0001	1.40		1.26–1.58		<0.0001
**High risk**	946	654	2.17		1.89–2.49		<0.0001	2.22		1.92–2.51		<0.0001
		**PCa**					**BPH**					
		**Blood samples**					**Blood samples**					
**Genotype^a^**		**MDA (nmol/mL)**		**GSH (nmol/mg** **protein)**		**ROS^#^**	**MDA (nmol/mL)**		**GSH (nmol/mg** **protein)**		**ROS^#^**	
**Low risk**		2.63±0.02		15.33±1.50		0.35±0.05	2.41±0.10		18.03±0.50		0.17±0.03	
**Intermediate risk**		2.94±0.07		11.32±0.80		0.73±0.11	2.67±0.02		13.32±0.20		0.32±0.06	
**High risk**		3.27±0.12		7.20±0.20		2.70±0.72	2.96±0.04		8.65±0.70		1.48±0.24	
		**Cells from urine sediments**					**Cells from urine sediments**					
**Genotype^a^**		**MDA (nmol/mL)**		**GSH (nmol/mg** **protein)**		**ROS^#^**	**MDA (nmol/mL)**		**GSH (nmol/mg** **protein)**		**ROS^#^**	
**Low risk**		2.83±0.05		18.00±1.50		0.38±0.02	2.55±0.03		21.29±1.00		0.16±0.06	
**Intermediate risk**		3.02±0.01		12.50±1.30		0.75±0.18	2.71±0.06		14.66±0.20		0.35±0.03	
**High risk**		3.62±0.14		7.10±0.90		2.80±1.52	3.22±0.08		9.06±0.30		1.52±0.12	

Low risk genotype: GSTP1Ile/Ile-PON1/192RR-PON1/55LL-CYP17A2A2-GLO1CC; Intermediate risk genotype: GSTP1Ile/Ile-PON1/192QQ-PON1/55LM-CYP17A1A2-GLO1CA; High risk genotype: GSTP1Ile/Val-PON1/192QR-PON1/55MM-CYP17A1A1-GLO1AA; BPH = benign prostatic hyperplasia, PCa = prostate cancer, *for age and PSA in logistic regression model. CI = confidence interval; OR = odds ratio. ^#^expressed as Rhodamine 123 fluorescence level; MDA: malonyldialdheyde; GSH: reduced glutathione; ROS: reactive oxygen species; ^a^p values are calculated using linear regression under an additive model, incorporating age and PSA as covariates; p<0.05.

### Multiple Comparisons

Multiple comparisons were necessary before an overall definitive statement about the significance of our findings could be made. All results, except those on the association of GLO1 polymorphism with PCa risk, withstood correction for multiple testing (p<0.05).

## Discussion

In the present study we evaluated, for the first time to our knowledge, the association between GLO1 −419C>A polymorphism and oxidative stress levels in PCa progression.

In fact, as previously suggested, the A to E substitution due to GLO1 SNP may determine a conformational modification in the enzyme, leading to an isoenzyme with a lower detoxification capacity [11]–[13]. As well known, GLO1 is an efficient antiglycation defence that decreases the concentration of reactive carbonyl compounds, such as MG, one of the most potent precursors of carbonyl stress-related AGEs [1]. Consequently, a decrease in the activity of this enzyme may result in an accumulation of AGEs and, in turn, of ROS and free radicals in human PCa cells. In fact, as well known, MG-derived AGEs are also very effective pro-oxidant molecules [5], [6] that importantly contribute to oxidative stress onset, a key event in PCa pathogenesis and progression [7]. Indeed, accumulation of AP, one of the most abundant MG-derived AGEs [3], [4], has been found in the present study, either in differently aggressive and invasive PCa cell lines models, or in biological fluids of patients with differently aggressive and invasive PCa, where such an accumulation well matched the presence of a less functioning GLO1 enzyme as well as a significant condition of oxidative stress. Moreover, MG-induced posttranslational adduction of selected target proteins [44], [45] is rapidly emerging as a novel mechanism of cancer cell survival signaling, a condition typically associated with cancer progression. Our results well correlate with those of Hermani et al. who demonstrated that the expression of the soluble form of AGEs receptor (sRAGE), which sensitively reflects AGEs burden [42], is enhanced and strongly associated with prostate carcinomas progression [46]. Besides, RAGE-AGEs interaction triggers activation of a multiple signaling pathways integrally linked to tumorigenesis [47] and essential for PCa development [48] and progression [49]. The results on the role of GLO1 polymorphism with oxidative stress in LNCaP and PC3 cell lines, and, above all, in the biological samples of patients with PCa at different grading and staging, suggested an important biological role of such enzyme and its polymorphism to the association with PCa progression. Therefore, it was reasonable to expect that the presence of a lower activity form of GLO1– the GLO1 A allele – might be predictive about severe consequences for an individual’s PCa risk of progression. In fact, we found that the mutant A allele conferred a dramatic risk of PCa progression compared to the wild C allele, either when BPH or healthy men controls were considered as the reference group. Less important was the association of GLO1 polymorphism with the risk of PCa development. In fact, we found that the mutant A allele conferred a modest risk of PCa occurrence compared to the wild C allele, again either when BPH or healthy men controls were considered as the reference group. Therefore, our results suggested a significant role for GLO1 in the progression rather than in the development of PCa. Recently, it has been demonstrated that also other SNPs in GLO1 locus result to be associated with GLO1 activity [13]. In the present exploratory study, we analyzed the common coding GLO1 −419C>A polymorphism, reasonably assuming that it might be more likely and directly correlated with GLO1 activity. In fact, it has been described that the substitution of Ala111Glu in GLO1 due to the-419C>A SNP, may cause conformational changes of the protein, very likely interfering with its 3D structure and affecting enzyme activity [14]. Certainly, we cannot rule out the possibility that other SNPs or number of tagSNPs from GLO1 locus, may affect the GLO1 enzymatic activity. Therefore, selection of additional SNPs is mandatory in future studies, in order to better evaluate the influence of genetic variations on PCa progression.

As to survival analysis, we found that GLO1 −419 A allele was significantly associated with an unfavourable survival prognosis in patients with localized (T1/T2) or low grade (GS 2–6) PCa. Therefore, if confirmed by additional studies, this polymorphism, preferably in combination with others, might help to differentiate between clinically significant and indolent prostate cancers and to predict the clinical course of early stages PCa. Unfortunately, the additional polymorphisms studied in the present work, either singly or in combination, did not show any significant association with PCa survival or progression. The combination was, however, significant in the association with PCa risk and positively correlated with the oxidative stress status shown by patients belonging to specific risk classes, thus providing biological plausibility. In fact, such genes encode proteins related to the control of oxidative stress, being able to detoxify free radicals (PON 1 and GSTP1) or reduce potential substrates for their production (CYP17). Similarly to GLO1, the polymorphic variants may determine conformational modifications leading to isoenzymes with an altered functional capacity and, consequently, contributing to the onset of a marked oxidative stress condition as, indeed, here, pointed out by the genetic association with blood or urinary levels of oxidative stress indices.

It is now well accepted that genetic association studies have helped enhance our understanding of the pathogenesis of human cancers, including PCa. However, establishing the clinical utility of these SNPs have proven challenging mainly because of the difficulties encountered in performing SNP analysis (lack of reproducibility, inadequate sample sizes, publication bias, genetic background) [50]. Another additional issue concerning SNPs analysis is the biological plausibility of genetic association. The chance that significant associations, even with impressive low p-values, are really true, depends largely on whether the association is biological plausible [51]. We would like to emphasize that the present work, providing biological plausibility to the association of GLO1 polymorphism with the progression or to the combination of all the analyzed polymorphisms with the risk of PCa, bring up a valid contribution to the study of genetic associations. Since a significant number of independent statistical tests was applied in the study, multiple comparisons were necessary before an overall definitive statement about the significance of our findings could be made. Except for the data on the already observed weak association of GLO1 polymorphism with PCa risk, that did not withstand correction for multiple testing, thus certainly suggesting further investigation, we found evidence against the null hypothesis (the *p* value stayed still significant <0.05 after correction for multiple testing using permutation-based methods) for all the other analyses, indicating the robustness of the results.

In conclusion, our study pointed out a significant role for GLO1 in PCa progression and that GLO1 −419A risk allele may be an independent prognostic factor for survival. Finally, we provided evidence of the biological plausibility of such polymorphism, either alone or in combination with other ones, all related to oxidative stress control that, as known, represents a key event in PCa development and progression. Since this is the first study examining the association between GLO1 −419C>A polymorphism and PCa, additional research is required.

## References

[1] Rabbani N, Thornalley PJ (2012) Methylglyoxal, glyoxalase 1 and the dicarbonyl proteome. Amino Acids 42: 1133–1142. 20963454 10.1007/s00726-010-0783-0

[2] Thornalley PJ, Rabbani N (2011) Glyoxalase in tumorigenesis and multidrug resistance. Semin Cell Dev Biol 22: 318–325. 21315826 10.1016/j.semcdb.2011.02.006

[3] Kim J, Kim OS, Kim CS, Sohn E, Jo K, et al (2012a) Accumulation of argpyrimidine, a methylglyoxal-derived advanced glycation end product, increases apoptosis of lens epithelial cells both in vitro and in vivo. Experimental and Molecular Medicine 44: 167–175. 22139526 10.3858/emm.2012.44.2.012 PMC3296813

[4] Kim KM, Kim YS, Jung DH, Lee J, Kim JS (2012b) Increased glyoxalase I levels inhibit accumulation of oxidative stress and an advanced glycation end product in mouse mesangial cells cultured in high glucose. Experimental Cell Research 318: 152–159. 22036650 10.1016/j.yexcr.2011.10.013

[5] Desai KM, Chang T, Wang H, Banigesh A, Dhar A, et al (2010) Oxidative stress and aging: is methylglyoxal the hidden enemy? Can J Physiol Pharmacol 88: 273–284. 20393592 10.1139/Y10-001

[6] Sena CM, Matafome P, Crisostomo J Rodrigues L, Fernandes R, et al (2012) Methylglyoxal promotes oxidative stress and endothelial dysfunction. Pharmacol Res 65: 497–506. 22425979 10.1016/j.phrs.2012.03.004

[7] Khandrika L, Kumar B, Koul S, Maroni P, Koul HK (2009) Oxidative stress in prostate cancer. Cancer Lett 282: 125–136. 19185987 10.1016/j.canlet.2008.12.011 PMC2789743

[8] Gupta-Elera G, Garrett AR, Robison RA, O’Neill KL (2012) The role of oxidative stress in prostate cancer. Eur J Cancer Prev 21: 155–162. 21857523 10.1097/CEJ.0b013e32834a8002

[9] Shiota M, Yokomizo A, Naito S (2011) Oxidative stress and androgen receptor signaling in the development and progression of castration-resistant prostate cancer. Free Radic Biol Med 51: 1320–1328. 21820046 10.1016/j.freeradbiomed.2011.07.011

[10] Acharya A, Das I, Chandhok D, Saha T (2010) Redox regulation in cancer: a double-edged sword with therapeutic potential. Oxid Med Cell Longev 3: 23–34. 20716925 10.4161/oxim.3.1.10095 PMC2835886

[11] Junaid MA, Kowal D, Barua M, Pullarkat PS, Sklower Brooks S, et al (2004) Proteomic studies identified a single nucleotide polymorphism in glyoxalase I as autism susceptibility factor. Am J Med Genet 131: 11–17. 15386471 10.1002/ajmg.a.30349 PMC1360505

[12] Barua M, Jenkins EC, Chen W, Kuizon S, Pullarkat RK, et al (2011) Glyoxalase I polymorphism rs2736654 causing the Ala111Glu substitution modulates enzyme activity–implications for autism. Autism Res 4: 262–270. 21491613 10.1002/aur.197 PMC3138858

[13] Peculis R, Konrade I, Skapare E, Fridmanis D, Nikitina-Zake L, et al (2013) Identification of glyoxalase 1 polymorphisms associated with enzyme activity. Gene 515: 140–143. 23201419 10.1016/j.gene.2012.11.009

[14] Wu JC, Li XH, Wang JB, Tang JF, Wang YF, et al (2011) Glyoxalase I and aldose reductase gene polymorphisms and susceptibility to carotid atherosclerosis in type 2 diabetes. Genet Test Mol Biomarkers 15: 273–279. 21294693 10.1089/gtmb.2010.0075

[15] Kalousová M, Jáchymová M, Germanová A, Kubena AA, Tesar V, et al (2010) Genetic predisposition to advanced glycation end products toxicity is related to prognosis of chronic hemodialysis patients. Kidney Blood Press Res 33: 30–36. 20185929 10.1159/000285845

[16] Williams R4th, Lim JE, Harr B, Wing C, Walters R, et al (2009) A common and unstable copy number variant is associated with differences in Glo1 expression and anxiety-like behavior. PLoS One 4: e4649 doi: 10.1371/journal.pone.0004649 19266052 10.1371/journal.pone.0004649 PMC2650792

[17] Sidoti A, Antognelli C, Rinaldi C, D’Angelo R, Dattola V, et al (2007) Glyoxalase I A111E, paraoxonase 1 Q192R and L55M polymorphisms: susceptibility factors of multiple sclerosis? Mult Scler 13: 446–453. 17463067 10.1177/13524585070130040201

[18] Antognelli C, Del Buono C, Ludovini V, Gori S, Talesa VN, et al (2009) CYP17, GSTP1, PON1 and GLO1 gene polymorphisms as risk factors for breast cancer: an Italian case-control study. BMC Cancer 9: 115 doi: 10.1186/1471-2407-9-115 19379515 10.1186/1471-2407-9-115 PMC2680904

[19] Krechler T, Jáchymová M, Mestek O, Zák A, Zima T, et al (2010) Soluble receptor for advanced glycation end-products (sRAGE) and polymorphisms of RAGE and glyoxalase I genes in patients with pancreas cancer. Clin Biochem 43: 882–886. 20398646 10.1016/j.clinbiochem.2010.04.004

[20] Meagher EA, FitzGerald GA (2000) Indices of lipid peroxidation in vivo: strengths and limitations. Free Radic Biol Med 28: 1745–1750. 10946216 10.1016/s0891-5849(00)00232-x

[21] Mearini E, Antognelli C, Del Buono C, Cochetti G, Giannantoni A, et al (2009) The combination of urine DD3(PCA3) mRNA and PSA mRNA as molecular markers of prostate cancer. Biomarkers 14: 235–243. 19489685 10.1080/13547500902807306

[22] Talesa VN, Antognelli C, Del Buono C, Stracci F, Serva MR, et al (2009) Diagnostic potential in prostate cancer of a panel of urinary molecular tumor markers. Cancer Biomark 5: 241–251. 20037200 10.3233/CBM-2009-0109 PMC12922823

[23] Jansson KF, Akre O, Garmo H, Bill-Axelson A, Adolfsson J, et al (2012) Concordance of tumor differentiation among brothers with prostate cancer. Eur Urol 62: 656–661. 22386193 10.1016/j.eururo.2012.02.032

[24] Ishak MB, Giri VN (2011) A systematic review of replication studies of prostate cancer susceptibility genetic variants in high-risk men originally identified from genome-wide association studies. Cancer Epidemiol Biomarkers Prev 20: 1599–1610. 21715604 10.1158/1055-9965.EPI-11-0312

[25] Varghese JS, Easton DF (2010) Genome-wide association studies in common cancers–what have we learnt? Curr Opin Genet Dev 20: 201–209. 20418093 10.1016/j.gde.2010.03.012

[26] Kim ST, Cheng Y, Hsu FC, Jin T, Kader AK, et al (2010) Prostate cancer risk-associated variants reported from genome-wide association studies: meta-analysis and their contribution to genetic variation. Prostate 70: 1729–1738. 20564319 10.1002/pros.21208 PMC3013361

[27] Antognelli C, Mearini L, Talesa VN, Giannantoni A, Mearini E (2005) Association of CYP17, GSTP1, and PON1 polymorphisms with the risk of prostate cancer. Prostate 63: 240–251. 15538743 10.1002/pros.20184

[28] Stark JR, Perner S, Stampfer MJ (2009) Gleason score and lethal prostate cancer: does 3+4 = 4+3? J Clin Oncol 27: 3459–3464. 19433685 10.1200/JCO.2008.20.4669 PMC2717753

[29] Basu A, Banerjee H, Rojas H, Martinez SR, Roy S, et al (2011) Differential expression of peroxiredoxins in prostate cancer: consistent upregulation of PRDX3 and PRDX4. Prostate 71: 755–65. 21031435 10.1002/pros.21292 PMC3107902

[30] Ferro E, Visalli G, Civa R, La Rosa MA, Randazzo Papa G, et al (2012) Oxidative damage and genotoxicity biomarkers in transfused and untransfused thalassemic subjects. Free Rad Biol Med 53: 1829–1837. 22995637 10.1016/j.freeradbiomed.2012.08.592

[31] Antognelli C, Mezzasoma L, Fettucciari K, Mearini E, Talesa VN (2013) Role of glyoxalase I in the proliferation and apoptosis control of human LNCaP and PC3 prostate cancer cells. Prostate 73: 121–132. 22653787 10.1002/pros.22547

[32] Mannervik B, Aronsson AC, Marmstal E, Tibellin G (1981) Glyoxalase I (rat liver). In: Jakoby WB: Methods in Enzymatic Analysis, 297–301. 10.1016/s0076-6879(81)77041-1 6173571

[33] Chavan SV, Chavan NR, Balaji A, Trivedi VD, Chavan PR (2011) A pilot study on the use of serum glyoxalase as a supplemental biomarker to predict malignant cases of the prostate in the PSA range of 4–20 ng/ml. Indian J Med Res 134: 458–462. 22089607 PMC3237243

[34] Fettucciari K, Fetriconi I, Mannucci R, Nicoletti I, Bartoli A, et al (2006) Group B Streptococcus induces Macrophage Apoptosis by Calpain Activation. J Immunol 176: 7542–7556. 16751401 10.4049/jimmunol.176.12.7542

[35] Raj DSC, Lim G, Levi M, Qualls C, Jain SK (2004) Advanced glycation end products and oxidative stress are increased in chronic allograft nephropathy. American Journal of Kidney Diseases 43: 154–160. 14712439 10.1053/j.ajkd.2003.09.021

[36] Szabados E, Fischer GM, Gallyas F, Kispal G, Sumegi B (1999) Enhanced ADP-ribosylation and its diminution by lipoamide after ischemia-reperfusion in perfused rat heart. Free Radic Biol Med 27: 1103–1113. 10569643 10.1016/s0891-5849(99)00151-3

[37] Guzel S, Kiziler L, Aydemir B, Alici B, Ataus S, et al (2012) Association of Pb, Cd, and Se concentrations and oxidative damage-related markers in different grades of prostate carcinoma. Biol Trace Elem Res 145: 23–32. 21809052 10.1007/s12011-011-9162-2

[38] Chen YC, Hu FJ, Chen P, Wu YR, Wu HC, et al (2010) Association of TNF-alpha gene with spontaneous deep intracerebral hemorrhage in the Taiwan population: a case control study. BMC Neurol 10: 41 doi: –––10.1186/1471–2377–10–41 20534169 10.1186/1471-2377-10-41 PMC2891694

[39] Larsen MH, Albrechtsen A, Thørner LW, Werge T, Hansen T, et al (2013) Genome-Wide Association Study of Genetic Variants in LPS-Stimulated IL-6, IL-8, IL-10, IL-1ra and TNF-α Cytokine Response in a Danish Cohort. PLoS One 8: e66262. 23823136 10.1371/journal.pone.0066262 PMC3688878

[40] Nakadate Y, Uchida K, Shikata K (2009) The formation of argpyrimidine, a methylglyoxal-arginine adduct, in the nucleus of neural cells. Biochem Biophys Res Commun 378: 209–212. 19014907 10.1016/j.bbrc.2008.11.028

[41] Nie J, Hou FF (2012) Role of reactive oxygen species in the renal fibrosis. Chin Med J (Engl) 125: 2598–25602. 22882945

[42] Kalousová M, Germanová A, Jáchymová M, Mestek O, Tesar V, et al (2008) A419C (E111A) polymorphism of the glyoxalase I gene and vascular complications in chronic hemodialysis patients. Ann N Y Acad Sci 1126: 268–271. 18079478 10.1196/annals.1433.012

[43] Politi P, Minoretti P, Falcone C, Martinelli V, Emanuele E (2006) Association analysis of the functional Ala111Glu polymorphism of the glyoxalase I gene in panic disorder. Neurosci Lett 396: 163–166. 16352396 10.1016/j.neulet.2005.11.028

[44] Sakamoto H, Mashima T, Yamamoto K, Tsuruo T (2002) Modulation of heat-shock protein 27 (Hsp27) anti-apoptotic activity by methylglyoxal modification. J Biol Chem 277: 45770–45775. 12226095 10.1074/jbc.M207485200

[45] Van Heijst JW, Niessen HW, Musters RJ, van Hinsbergh VW, Hoekman K, et al (2006) Argpyrimidine-modified heat shock protein 27 in human non-small cell lung cancer: a possible mechanism for evasion of apoptosis. Cancer Lett 241: 309–319. 16337338 10.1016/j.canlet.2005.10.042

[46] Hermani A, De Servi B, Medunjanin S, Tessier PA, Mayer D (2006) S100A8 and S100A9 activate MAP kinase and NF-kappaB signaling pathways and trigger translocation of RAGE in human prostate cancer cells. Exp Cell Res 312: 184–197. 16297907 10.1016/j.yexcr.2005.10.013

[47] Gebhardt C, Gebhardt C, Riehl A, Durchdewald M (2008) RAGE signaling sustains inflammation and promotes tumor development. J Exp Med 205: 275–285. 18208974 10.1084/jem.20070679 PMC2271015

[48] Ishiguro H, Nakaigawa N, Miyoshi Y, Fujinami K, Kubota Y, et al (2005) Receptor for advanced glycation end products (RAGE) and its ligand, amphoterin are overexpressed and associated with prostate cancer development. Prostate 64: 92–100. 15666359 10.1002/pros.20219

[49] Allmen EU, Koch M, Fritz G, Legler DF (2008) V domain of RAGE interacts with AGEs on prostate carcinoma cells. Prostate 68: 748–758. 18302220 10.1002/pros.20736

[50] Colhoun HM, McKeigue PM, Davey Smith G (2003) Problems of reporting genetic associations with complex outcomes. Lancet 361: 865–872. 12642066 10.1016/s0140-6736(03)12715-8

[51] Van der Velden WJ, Feuth T, Stevens WB, Donnelly JP, Blijlevens NM (2011) Bone Issues in genetic association studies: limitations of statistical analysis and biological plausibility. Marrow Transplant 46: 906–907. 10.1038/bmt.2010.211 20818447

